# Association between lower extremity physical function and physical activity after ischemic stroke: Longitudinal findings from the MOBITEC-Stroke project

**DOI:** 10.1177/20503121241281147

**Published:** 2024-10-09

**Authors:** Christoph Jäger, Michelle Ryan, Nikki Rommers, Janine Schär, Robert Weibel, Reto W Kressig, Arno Schmidt-Trucksäss, Stefan Engelter, Nils Peters, Timo Hinrichs, Roland Rössler

**Affiliations:** 1 University Department of Geriatric Medicine Felix Platter, University of Basel, Basel, Switzerland; 2 Department of Clinical Research, University of Basel, University Hospital Basel, Basel, Switzerland; 3 Neurology and Stroke Center, Klinik Hirslanden, Zurich, Switzerland; 4 Department of Geography, University Research Priority Program Dynamics of Healthy Aging, University of Zurich, Zurich, Switzerland; 5 Division of Sport and Exercise Medicine, Department of Sport, Exercise, and Health, University of Basel, Basel, Switzerland; 6 Department of Neurology and Stroke Center, University Hospital Basel, University of Basel, Basel, Switzerland

**Keywords:** Stroke, physical activity, lower extremity physical function, timed up-and-go test, mobility, rehabilitation

## Abstract

**Background:**

Stroke often results in physical impairments. Physical activity is crucial for rehabilitation, enhancing mobility, strength, and overall health. This study examines the association between Timed Up-and-Go (TUG) test performance and changes in physical activity to improve lower extremity physical function.

**Methods:**

The MOBITEC-Stroke Cohort Study (“Recovery of mobility function and life-space mobility after ischemic stroke”) included patients with a first incidence of stroke. Data assessed 3 and 12 months after stroke were used for analysis. Linear regression model adjusted for age, sex, instrumental activities of daily living, Falls Efficacy Scale-International, modified Ranking Scale, and National Institutes of Health Stroke Scale-score was used to examine the relationship between lower extremity physical function (i.e., TUG) and change in physical activity (i.e., minutes of physical activity measured with a wrist-worn accelerometer over 1 week).

**Results:**

Longitudinal data of 49 patients (65% male, mean age 71.2 (SD: 10.4) years) were analyzed. Mean daily physical activity was 291.6 (SD: 96.2) min at 3 months and 298.9 (SD: 94.4) min at 12 months, with a change from 3 to 12 months of 7.3 min (95% CI: −9.4 to 24.0; *p* = 0.394) post-stroke. We observed significant relationships between the baseline TUG performance and the change in total physical activity over 9 months (*p* = 0.011) and between the change of TUG performance over time and the change in total physical activity (*p* = 0.022).

**Conclusion:**

Our findings indicate that better initial lower extremity physical function and higher improvements in function over time are associated with a greater increase in physical activity levels after stroke. This suggests that interventions aimed at maintaining and improving lower extremity physical function may positively affect physical activity levels.

## Introduction

Stroke is a leading global cause of disability and mortality, often resulting in a wide range of physical impairments and functional limitations.^1^ Physical activity (PA) encompasses several aspects of an individual’s daily activities and bodily movements,^2^ with accelerometer-based devices commonly employed to quantify and characterize PA in research.^3^

Crucially, PA emerges as a cornerstone in stroke rehabilitation, offering numerous benefits for stroke survivors such as enhanced mobility, muscle strength, and cardiovascular health.^4,5^ Studies suggest that light PA can significantly improve secondary prevention outcomes, impacting factors such as blood pressure, serum lipoprotein ratio, and cholesterol levels.^6,7^

Although the importance of post-stroke PA is well-recognized and documented in guidelines,^8^ many stroke survivors face challenges in maintaining regular PA.^9,10^ Identifying barriers and facilitators to post-stroke PA is essential for optimizing rehabilitation and long-term outcomes. Early identification of individuals at risk for low PA during rehabilitation can address specific needs, reduce risks of recurrent strokes,^11^ and enhance overall quality of life.^12^

Approximately 80% of stroke survivors experience motor disability,^1^ and two-thirds face challenges in walking and maintaining balance,^13^ both integral parts of lower extremity physical function (LEPF). A meta-analysis has highlighted a positive association between LEPF and key measures such as the 6-min walking test, gait speed, balance, and post-stroke PA.^14^ However, the studies included were cross-sectional, and PA levels were mainly assessed via self-report. Practical, objective assessments to evaluate LEPF in clinical settings are needed, and the Timed Up-and-Go (TUG) test^15^ is a feasible, reliable tool for evaluating LEPF.^16^ An important priority in current stroke research is determining the most effective assessments to evaluate the abilities necessary for everyday life after stroke.^17^

This study addresses this research priority by investigating the association between TUG performance at baseline (i.e., 3 months post-stroke) and the changes in TUG performance over time with changes in PA during the same period.

## Methods

### Design and population

This study is a secondary analysis of data from the MOBITEC-Stroke study. MOBITEC-Stroke (“Recovery of mobility function and life-space mobility after ischemic stroke”; ISRCTN85999967) was a prospective cohort observational study. Assessments were performed at four distinct intervals following a stroke: at 3 months (T0), 6 months (T1), 9 months (T2), and 12 months (T3). In addition, each subject’s clinical data from their initial admission to the Stroke Center (i.e., shortly after the stroke event) were available. For the present analyses, data from the 3-month and the 12-month assessment were used. The study received approval by the ethical committee of Northwestern and Central Switzerland (project-ID: 2019-00989). All participants provided written informed consent. Additional details are described in the study protocol.^18^ In addition to the present study, another research question emerged from the main study, focusing on quality of life.^19^

Inclusion criteria for the MOBITEC-Stroke study were as follows: first ischemic stroke within 3 months before the study; age ⩾18 years; capable of verbal communication; full capability to give written informed consent; and capability to get out of a chair on their own and the ability to walk on their own for 20 m at their own pace without help from another person. To be included in the study, a participant was required to have one or more of the following stroke-related symptoms, which may affect mobility: visual field defect or disturbance, stance/gait ataxia, central vestibular dysfunction, lower limb paresis, or attentional deficit.

Exclusion criteria were as follows: living in an assisted living facility or nursing home; being unable to walk without assistance (modified Rankin Scale > 3); having a serious cognitive deficit (Montreal Cognitive Assessment score < 21 or, <20 for persons with 12 years of education or less)^20^; being terminally ill; having undergone lower limb orthopedic surgery within a year before the study; having undergone rehabilitation after surgery around the time of the stroke; having depression or other acute psychiatric illness; and self-report of significant difficulty walking or climbing stairs pre-stroke.

Patients at the University Hospital Basel’s Stroke Center with an acute ischemic stroke between October 2019 and March 2021 were evaluated for eligibility and, if eligible, offered participation. A formal a priori sample size estimation was conducted aimed at the main outcome (normal walking speed) of the project. The sample size was determined using simulations to estimate the required number of participants for a linear mixed-effects model assessing 10-m habitual walking speed over four time points. The analysis aimed to detect an overall trend and clinically significant differences of 0.14 m/s between adjacent time points, assuming a standard deviation of 0.3 m/s. Simulations were conducted for correlations of 0.2, 0.5, 0.6, and 0.7 between time points, with 5000 repetitions each. The tests were two-sided with a 5% significance level and 80% power. To account for a 5% dropout rate per time point, sample sizes were adjusted. Based on a plausible correlation of at least 0.6, we decided to recruit 59 subjects.^18^

### Variables and measurements

#### Outcomes of interest

The outcome of interest of the study at hand was total PA (i.e., minutes per valid measurement day), as recent studies showed that not only moderate or vigorous PA but also light PA has a beneficial effect on cardiovascular health post-stroke.^21,22^ At 3 and 12 months after the stroke, PA was evaluated using a wrist-worn accelerometer (GeneActiv, Activinsights Ltd., Kimbolton, UK) positioned on the non-paretic side (or, for patients without a paresis, on the non-dominant wrist).^23^ All participants were asked to wear the device continuously over seven full days according to the guidelines for the measurement of PA after stroke.^24^ The GeneActiv is a triaxial, ±6 g seismic acceleration sensor measuring and storing data in SI units (m/s^2^). Accelerometer data were converted into the metabolic equivalent of task (MET) using the protocol by Esliger et al.^23^ In short, they synchronized accelerometer data during various activities with the measurement of V’O_2_. These measured V’O_2_ values were then converted to METs using V’O_2_ standard conversion of 1 MET = 3.5 ml kg^−1^ min^−1^ and then coded into the categories sedentary (<1.5 METs), light (105–3.99 METs), moderate (4.0–6.99 METs), and vigorous (>7 METs) activity. Minutes spent in light, moderate, and vigorous activity were summed to obtain total PA. Raw data processing was performed in R using the GGIR package version 3.0.^25^ A valid measurement day was defined as one having at least 12 h of recording time. To be included in the analysis, each recording session was required to contain data from at least four valid days, including at least one weekend day.

#### Independent variable of interest

The TUG test was performed to measure LEPF.^15^ The TUG test is a commonly used clinical assessment tool to evaluate a person’s mobility and risk of falling. Participants were asked to rise from a chair, walk 3 m around a cone, and back to the chair to sit down again. Using the armrest and/or walking aid was tolerated. The performance, defined as the required time in seconds, was measured by trained assistants using a stopwatch. The timing started with the command given by the assessor and stopped when the participant had seated contact with the chair again. Lower TUG times indicate better LEPF.

#### Further independent variables

The Falls Efficacy Scale – International version (FES-I)^26^ is a questionnaire designed to measure individuals’ confidence in carrying out daily activities without experiencing a fall. The scale consists of 16 items, with each item rated on a scale from 1 (not at all concerned) to 4 (very concerned). Hence, the final score can range from 16 to 64, with higher scores indicating greater fear of falling.^27^

The National Institutes of Health Stroke Scale (NIHSS) is a tool used for assessing the severity of neurological impairment in individuals who have experienced a stroke and guiding treatment decisions.^28^ The total score can range from 0 (indicating no neurological deficits) to 42 (indicating severe neurological impairment).

The modified Rankin Scale (mRS)^29^ is a commonly used tool for assessing and quantifying the degree of disability or functional impairment in individuals who have experienced a stroke or other neurological conditions to determine their level of disability. The mRS categorizes an individual’s functional status based on their level of disability or independence. The scale typically ranges from 0 (no symptoms at all) to 6 (dead), with each level corresponding to a specific degree of disability.

Age and sex were obtained from medical records. Difficulties in Instrumental Activities of Daily Living (IADL)^30^ were assessed by self-report. The 8-item questionnaire includes categories such as housekeeping, preparing food, shopping, doing laundry, handling medication and finances, and using the telephone and transportation. In each category, the item description that most closely resembles the participants’ highest functional level has to be chosen (e.g., “answers telephone” = 1 or “does not use telephone at all” = 0). A total score of 0 indicates low, and a score of 8 high functioning.^31^

### Statistical analysis

Participant characteristics were summarized using descriptive statistics. For numerical variables, we described normally distributed variables using the mean and standard deviation, while non-normally distributed variables were reported using the median and interquartile range (IQR). Categorical variables were represented by frequency and percentage.

The relationship between LEPF, as measured by TUG performance, at baseline (3 months post-stroke), the change in LEPF (from 3 to 12 months post-stroke), and the change in PA was examined using linear regression analysis. All models were adjusted for age category (i.e., median split: age ⩽ 74.3 years and age > 74.3 years), IADL score, sex, FES-I score, mRS (dichotomized; 0–1 and 2–3), and NIHSS score (dichotomized; 0–1 and ⩾2). TUG time was log-transformed prior to the analysis due to the skewed distribution.

Participants with missing outcome data (i.e., PA) were excluded from all analyses (i.e., no imputation in the outcome variable was conducted). Missing values for predictor variables (i.e., one missing TUG observation and two NIHSS scores) were assumed to be missing at random. These three values were imputed using Multiple Imputation by Chained Equations (MICE) with the “mice” package (version 3.15.0)^32^ in R (version 4.2.1)^33^ alongside RStudio (version 2022.07.2).^34^

The “tidyverse” package (version 2.0)^35^ was used for data management. We generated 60 datasets through the imputation process. For imputing continuous variables, predictive mean matching was applied, while logistic or multinomial regression was used for categorical variables. Using each of the 60 imputed datasets, we performed the linear regression analysis, and results were combined following Rubin’s rules,^36^ providing us with mean estimates and standard deviations for our model parameters.

## Results

Six out of the 59 participants dropped out between the 3-month and the 12-month assessment. Reasons for the dropout were health-related (*n* = 3), lack of interest (*n* = 2), and fear of infection with COVID-19 (*n* = 1). Details about the participant flow through the study have been described elsewhere.^37^ Regarding the GeneActiv measurement, we had four missing values due to technical problems (i.e., battery failure notion or corrupt data file) or devices that were lost by participants. Accordingly, data from 49 community-residing ambulatory patients (32 males and 17 females) were analyzed. The mean recording period was 6.76 (SD: 0.83) days, and each participant’s data included at least one weekend day. A total of 87% of the recorded data points reflected a full 24 h of continuous GeneActiv wear time per day, meaning there were no measuring gaps when the device was not worn. Every day included in the analysis had at least 12 h of data. The average daily non-wear time was 20.82 (SD: 95.23) minutes. Characteristics of participants at baseline (i.e., 3 months after stroke) are specified in Table 1. Notably, the NIHSS, mRS, IADL, and FES-I scores are all on the lower scale.

**Table 1.** Baseline characteristics of the study participants (i.e., 3 months post-stroke).

Variable	Value	Missing (*N*)
Number of participants (*N*)	49	
Sex = male (*N* (%))	32 (65.3)	
Age (years, mean (SD))	71.2 (10.4)	
Living alone (*N* (%))	25 (51.0)	
Education (years, mean (SD))	13.5 (3.5)	
Influence of finances on life (*N* (%))		
“Has somewhat complicated my life”	3 (6.1)	
“Has significantly complicated my life”	1 (2.0)	
“Had no influence”	45 (91.8)	
NIHSS score (0–42, lower is better) (median (IQR))	2.0 (1.0)	2
mRS (0–6, lower is better) (median (IQR))	1.0 (1.0)	
IADL (8–32, lower is better) (median (IQR))	9.0 (8.0, 11.0)	
FES-I (16–64, lower is better) (mean (SD))	20.9 (5.9)	
TUG time (s) (mean (SD))	10.0 (4.3)	1

*N*: number of participants; SD: standard deviation; IQR: interquartile range; NIHSS: National Institutes of Health Stroke Scale; mRS: modified Rankin Scale; IADL: Instrumental Activity of Daily Living; TUG: Timed Up-and-Go test; FES-I: Falls Efficacy Scale International version.

Table 2 shows the TUG time in seconds and the total PA over the 7-day period in min at 3 and 12 months after stroke and their change within these 9 months.

**Table 2.** Timed Up-and-Go performance and total physical activity over time (*N* = 49) and their change over time with corresponding 95% CI and *p*-value.

Variable	3 months	12 months	Change (i.e., 12–3 months)		
	Mean (SD)	Mean (SD)	Mean (SD)	95%CI	*p*-Value
PA (min/d)	291.6 (96.2)	298.9 (94.4)	7.31 (59.6)	−9.4 to 24.0	0.395
TUG (sec)	10.0 (4.3)	8.8 (2.9)	−1.26 (3.4)	−2.2 to −0.3	0.011

PA: total daily physical activity; SD: standard deviation; CI: confidence interval; TUG: Timed Up-and-Go test.

Our regression analysis examined the relationship between TUG performance and change in PA. We considered both the baseline TUG time (log score) and the change in TUG time (log score) over the study period. For both the log TUG time at baseline and the change in the log TUG time (i.e., from 3 to 12 months post-stroke), we observed significant negative associations with the change score of PA (Table 3).

**Table 3.** Results of regression models investigating the relation of Timed Up-and-Go time (i.e., at baseline and change over time) and change in physical activity (*N* = 49).

Variable	Estimate	Lower 95% CI	Upper 95% CI	*p*-Value
TUG time baseline (log score)	−90.346	−155.841	−24.851	0.011
TUG time change (log score)	−108.293	−186.148	−30.438	0.022

The model was adjusted for age category (i.e., median split), sex, National Institute of Health Stroke Scale (NIHSS) score, modified Rankin Scale (mRS) score, Instrumental Activities of Daily Living (IADL), and Falls Efficacy Scale – International versionfficacy (FES-I) score.

TUG: timed up-and-go test; CI: confidence interval.

To illustrate the association with PA, we present two examples from our analysis: (1) A 10% faster TUG test time at baseline (i.e., participants who completed the TUG test 10% faster than the average time of all participants at baseline) was associated with an increase of 9.5 min (95% CI: 2.6–16.4) in daily PA compared to participants with the average TUG test time. 2) Over the study period, a 10% improvement in TUG test performance (i.e., a 10% decrease in the time taken to complete the TUG test compared to their baseline performance) corresponded to an additional 11.4 min (95% CI: 3.2–19.6) of daily PA.

## Discussion

### Main findings

The results of this study provide insight into LEPF and total PA in a cohort of stroke survivors. The analysis focused on patients at two distinct time points: the baseline assessment, conducted 3 months after the stroke event, and the follow-up assessment at 12 months post-stroke. Comparison between these time points provided valuable insights into the dynamic nature of PA recovery. We observed that better TUG performances (i.e., shorter time to complete the test) at baseline were associated with a greater improvement in total PA during the study period. Furthermore, we observed that an improvement in TUG performance from 3 to 12 months was linked to an increase in total PA throughout the study. The change in total PA over the study period was not statistically significant. The scores for NIHSS, mRS, IADL, and FES-I are all located in the lower quartile of their specific range, indicating a sample relatively mobile and mildly affected by motor impairments, which might originate in the inclusion criteria for the study.

### Comparison with other studies

A recent study revealed gait speed and balance, both components of LEPF, to correlate with PA.^38^ Our data align with these findings, demonstrating that better TUG performance at baseline is linked to greater improvements in PA over time. It underscores the importance of LEPF in influencing PA levels in stroke survivors.

Ng et al.^39^ found the TUG test to also be a predictor of falls after stroke rehabilitation. Their results suggest that individuals with poorer LEPF or those experiencing a decline in mobility (i.e., comfortable gait speed) may engage in less PA. It highlights the interplay between physical function and engagement in regular PA. While our study supports that LEPF is an important factor, the lack of significant change in total PA prompts a more nuanced interpretation.

Two meta-analyses have yielded heterogeneous results regarding the relationship between PA after stroke and non-modifiable variables such as age and sex, while falls self-efficacy, TUG performance, and quality of life have been predominantly associated with a more physically active lifestyle among stroke survivors.^14,40^ However, the majority of the studies included in these meta-analyses were cross-sectional, and PA was assessed either via self-report or with a step counter. The findings from our longitudinal analysis with quantitative measurement of PA align with these studies. Our study contributes to a more comprehensive insight into the interplay between LEPF and PA as proposed by Thilarajah et al.^14^

A recent qualitative study highlighted the need for additional support and guidance for stroke survivors to maintain PA,^41^ but clinical settings often lack the time for comprehensive patient education. To improve secondary prevention, it is crucial to identify patients at risk of low PA engagement, allowing for targeted use of limited resources in rehabilitation. Although the TUG test is already a well-established tool in clinical settings, we want to emphasize its value in identifying individuals at risk of low PA. By incorporating the TUG test into routine evaluations, clinicians might be able to more accurately identify such individuals, tailor rehabilitation goals, and enhance individualized care. Our data support the implementation of the TUG test to effectively identify patients at risk and might enable clinicians then to implement personalized rehabilitation strategies. This approach allows healthcare providers to better counsel stroke survivors and their families on the importance of maintaining and improving LEPF and mobility, which can positively impact PA levels.

Future studies could build on these findings by investigating the role of wearable technology and digital health tools in monitoring TUG performance and PA, which could provide real-time data, allowing for timely interventions. Examining the impact of personalized rehabilitation programs based on TUG performance, incorporating both physical and psychosocial elements, may offer insights into optimizing patient outcomes.

### Strengths and limitations

This study’s strengths were its longitudinal design, which involved repeating measurements at predetermined intervals in our hospital’s strictly controlled laboratory setting, objective and validated PA measurement, and comprehensive statistical analysis. This study also has some limitations: despite a low dropout rate of just over 10%, the usable sample size was relatively small due to several cases of data loss. In addition, the study took place during the COVID-19 pandemic, so occasions and/or motivation for PA might have been limited to a certain extent. The average mRS of 1.53 and the average NIHSS score of 1.9 show that our sample was relatively mobile and that participant impairment was mild. This limits the generalizability of our study to stroke survivors who are only slightly affected.

## Conclusion

The relationship between LEPF (TUG time) and PA emphasizes the significance of addressing functional limitations in stroke survivors. It highlights the potential of the TUG test as a valuable tool for promoting individualized rehabilitation goals in stroke survivors. Further research on the topic with larger samples is indicated to test interventions that focus on function-enhancing exercises and therapies to improve functional capacity and promote PA engagement.

## Supplemental Material

sj-pdf-1-smo-10.1177_20503121241281147 – Supplemental material for Association between lower extremity physical function and physical activity after ischemic stroke: Longitudinal findings from the MOBITEC-Stroke project Supplemental material, sj-pdf-1-smo-10.1177_20503121241281147 for Association between lower extremity physical function and physical activity after ischemic stroke: Longitudinal findings from the MOBITEC-Stroke project by Christoph Jäger, Michelle Ryan, Nikki Rommers, Janine Schär, Robert Weibel, Reto W Kressig, Arno Schmidt-Trucksäss, Stefan Engelter, Nils Peters, Timo Hinrichs and Roland Rössler in SAGE Open Medicine

sj-pdf-2-smo-10.1177_20503121241281147 – Supplemental material for Association between lower extremity physical function and physical activity after ischemic stroke: Longitudinal findings from the MOBITEC-Stroke project Supplemental material, sj-pdf-2-smo-10.1177_20503121241281147 for Association between lower extremity physical function and physical activity after ischemic stroke: Longitudinal findings from the MOBITEC-Stroke project by Christoph Jäger, Michelle Ryan, Nikki Rommers, Janine Schär, Robert Weibel, Reto W Kressig, Arno Schmidt-Trucksäss, Stefan Engelter, Nils Peters, Timo Hinrichs and Roland Rössler in SAGE Open Medicine
