# Supplementary material for: Association between lower extremity physical function and physical activity after ischemic stroke: Longitudinal findings from the MOBITEC-Stroke project
Source: SAGE Open Med. 2024 Oct 9;12:20503121241281147. doi: 10.1177/20503121241281147 (PMC11504066; doi:10.1177/20503121241281147)
Supplement: sj-pdf-1-smo-10.1177_20503121241281147 – Supplemental material for Association between lower extremity physical function and physical activity after ischemic stroke: Longitudinal findings from the MOBITEC-Stroke project [file sj-pdf-1-smo-10.1177_20503121241281147.pdf]

SUPPLEMENTARY DATA

Translators'/Interviewers' notes for FES-I

The text of the FES-I below is the final version agreed by the authors on completion of the development study, prior to subsequent translation and validation in different languages. It became clear during the process of translation that there was no wording of the questionnaire that would translate easily into every EC language using exactly the same words and phrases. Consequently, these notes are intended to assist translators of the FES-I to express the same *meaning* of items, even if they cannot use quite the same words in their language. They may also assist interviewers who are asked for clarification of the meaning of items when the FES-I is administered by interview.

Instructions
Participants should answer items thinking about how they usually do the activity – for example, if they usually walk with an aid they should answer items about walking to show how concerned they would be about falling when using that aid. Some translators may find it helpful to clarify in the instructions (after the sentence on circling an opinion) 'The opinions you can choose from are: 1 = not at all concerned 2 = somewhat concerned 3 = fairly concerned 4 = very concerned.' In some languages it is better to translate the word 'opinion' as 'statement'.

Response categories
The word 'concerned' expresses a cognitive or rational disquiet about the possibility of falling, but does not express the emotional distress that would be expressed by terms such as 'worried', 'anxious' or 'fearful'. It is important to use a similar unemotional term, as respondents may be less willing to admit to emotions, which might be viewed as signs of weakness.

Item 3. In some EC languages 'simple' meals are best translated as 'everyday' meals, but the intention is to refer to a meal that does not require complex preparation, rather than one that is prepared every day.

Item 5. This item is intended to refer to shopping that is not extensive or recreational. In some languages the best translation is 'shopping for groceries'.

Item 7. This item refers to *any* stairs, not necessarily the flight of stairs in one's own house.

Item 8. In some languages 'neighbourhood' may be difficult to translate, and so 'walking around outside' can be used instead.

Item 12. In some languages it is necessary to add the term 'acquaintances' to friends and relatives, since this is a more common and casual category of relationship than friends. (see also comment on items 12, 13 and 16 below)

Item 13. 'Crowds' can be translated as 'many people' if necessary. (see also comment on items 12,13 and 16 below)

Item 14. It was found to be necessary to give examples of what is meant by uneven ground, but no examples could be found that were appropriate for all countries. Consequently, translators should choose any TWO examples from the following: cobblestones; poorly maintained pavement; rocky ground; unpaved surface.

Items 12, 13, 16. These items contain a greater element of ambiguity than many of the items assessing functional capabilities, because the physical activities involved in these social events may differ greatly for different respondents. However, it was decided that this ambiguity was acceptable because it is important to assess effects of fear of falling on social activities.

Now we would like to ask some questions about how concerned you are about the possibility of falling. For each of the following activities, please circle the opinion closest to your own to show how concerned you are that you might fall if you did this activity. Please reply thinking about how you usually do the activity. If you currently don't do the activity (e.g. if someone does your shopping for you), please answer to show whether you think you would be concerned about falling IF you did the activity.

		Not at all concerned 1	*Somewhat concerned 2*	*Fairly concerned 3*	*Very concerned 4*
1	Cleaning the house (e.g. sweep, vacuum or dust)	*1*	*2*	*3*	*4*
2	Getting dressed or undressed	*1*	*2*	*3*	*4*
3	Preparing simple meals	*1*	*2*	*3*	*4*
4	Taking a bath or shower	*1*	*2*	*3*	*4*
5	Going to the shop	*1*	*2*	*3*	*4*
6	Getting in or out of a chair	*1*	*2*	*3*	*4*
7	Going up or down stairs	*1*	*2*	*3*	*4*
8	Walking around in the neighbourhood	*1*	*2*	*3*	*4*

9	Reaching for something above your head or on the ground	*1*	*2*	*3*	*4*
10	Going to answer the telephone before it stops ringing	*1*	*2*	*3*	*4*
11	Walking on a slippery surface (e.g. wet or icy)	*1*	*2*	*3*	*4*
12	Visiting a friend or relative	*1*	*2*	*3*	*4*
13	Walking in a place with crowds	*1*	*2*	*3*	*4*
14	Walking on an uneven surface (e.g. rocky ground, poorly maintained pavement)	*1*	*2*	*3*	*4*
15	Walking up or down a slope	*1*	*2*	*3*	*4*
16	Going out to a social event (e.g. religious service, family gathering or club meeting)	*1*	*2*	*3*	*4*
