# Supplementary material for: Association between lower extremity physical function and physical activity after ischemic stroke: Longitudinal findings from the MOBITEC-Stroke project
Source: SAGE Open Med. 2024 Oct 9;12:20503121241281147. doi: 10.1177/20503121241281147 (PMC11504066; doi:10.1177/20503121241281147)
Supplement: sj-pdf-2-smo-10.1177_20503121241281147 – Supplemental material for Association between lower extremity physical function and physical activity after ischemic stroke: Longitudinal findings from the MOBITEC-Stroke project [file sj-pdf-2-smo-10.1177_20503121241281147.pdf]

1) **How many years did your education take?**

___ years

2) **You live in your household...**

o alone

o with at least one other person

3) **In the last four weeks, has your participation in social life been hindered by a problematic financial situation (e.g., lack of money, absence of state support)? Please refer to your desired level of social life.**

o Somewhat complicated my life.

o Significantly complicated my life.

o Had no influence.
